# Identification of Response Options to Artisanal and Small-Scale Gold Mining (ASGM) in Ghana via the Delphi Process

**DOI:** 10.3390/ijerph120911345

**Published:** 2015-09-10

**Authors:** Avik Basu, Sean Phipps, Rachel Long, George Essegbey, Niladri Basu

**Affiliations:** 1 School of Natural Resources and the Environment, University of Michigan, Ann Arbor, MI 48109, USA; E-Mail: abasu@umich.edu; 2 Faculty of Agricultural and Environmental Sciences, McGill University, CINE BuildingMacdonald Campus of McGill University, 21111 Lakeshore Rd., Ste. Anne de Bellevue, QC H9X 3V9, Canada; E-Mail: sean.phipps@mail.mcgill.ca; 3 Department of Environmental Health Sciences, University of Michigan School of Public Health, Ann Arbor, MI 48109, USA; E-Mail: rachlong@umich.edu; 4 Science and Technology Policy Research Institute, CSIR-STEPRI, P.O. Box CT. 519, Cantonments, Accra, Ghana; E-Mail: goessegbey@hotmail.com

**Keywords:** Delphi technique, Ghana, small-scale gold mining, policy development, consensus, questionnaires, survey method

## Abstract

The Delphi technique is a means of facilitating discussion among experts in order to develop consensus, and can be used for policy formulation. This article describes a modified Delphi approach in which 27 multi-disciplinary academics and 22 stakeholders from Ghana and North America were polled about ways to address negative effects of small-scale gold mining (ASGM) in Ghana. In early 2014, the academics, working in disciplinary groups, synthesized 17 response options based on data aggregated during an Integrated Assessment of ASGM in Ghana. The researchers participated in two rounds of Delphi polling in March and April 2014, during which 17 options were condensed into 12. Response options were rated via a 4-point Likert scale in terms of benefit (economic, environmental, and benefit to people) and feasibility (economic, social/cultural, political, and implementation). The six highest-scoring options populated a third Delphi poll, which 22 stakeholders from diverse sectors completed in April 2015. The academics and stakeholders also prioritized the response options using ranking exercises. The technique successfully gauged expert opinion on ASGM, and helped identify potential responses, policies and solutions for the sector. This is timely given that improvement to the ASGM sector is an important component within the UN Minamata Convention.

## 1. Introduction

### 1.1. Development

Originally, the tool of Cold War futurists and technology experts, the Delphi technique has gone on to enjoy a rich second life as a decision-making tool for both academia and public policy. Created in the 1950s by the RAND Corporation to provide forecasting on future military and technological developments for the United States Air Force [1,2], the technique sought to facilitate consensus among a team of experts as a means of making long-term predictions about complex situations [2]. To do so, the Delphi process relies on several rounds of anonymous polling with controlled feedback, still the core of most Delphi-style analyses today [2,3]. The primary benefit of this method of consensus building, according to its original framers, was to mitigate the psychological pressures of majority opinion and force participants to consider and explore new ways of thinking [2]. Very much in keeping with the mood of the time, the inventors of the Delphi technique sought to harness the power of group decision-making, standardize it, and modernize it, freeing it of the limitations of social pressures. Luckily, the technique has continued to grow beyond its developers' original intentions to become a more diverse and nuanced method of facilitating group decision-making.

### 1.2. Description of Techniques

The Delphi technique in its simplest form consists of multiple rounds of questionnaires given to a group of pre-selected “experts” [2]. The first round is normally open, allowing the participants to voice their opinions and determine priorities, while subsequent rounds are used to highlight areas of mutual concern and, eventually, reach a consensus [2]. Throughout the process, participants are provided with feedback about their fellow participants’ responses, whose identities are normally kept anonymous [4]. The questionnaires are used to collect data around a question in the form of comments or ranking scales [3]. This data is then fed back to the panel in quantitative form [3]. Participants are expected to re-consider their opinions within the context of the group, and if they feel necessary, modify them [5]. The hope is that eventually a consensus will be reached among the participants that reflects expert opinion on the subject [5]. 

### 1.3. Evolution of Technique

Following its use by the RAND Corporation, the Delphi technique was increasingly adopted for civilian use. According to Reiger, the technique spread to the business and academic realm throughout the 1960s, reaching the height of its popularity in the 1970s, before declining due to concerns about the validity of its methodology [6]. Since then its popularity has increased somewhat, with a number of different forms of Delphi-style techniques being developed to adapt to a wider field of disciplines and purposes, as awareness increases of the different strengths and weaknesses of the method as a forecasting technique [7,8].

The Delphi technique today exists in a number of different forms, and spans a wide array of applications. Most notably the emphasis on consensus has declined, with users instead relying on it as merely a reliable measure of group opinion over time [9]. Other innovations include the disaggregative Delphi, which measures clusters of opinion or dissensus rather than consensus [10], Delphi poll directed towards policy creation [7], and internet-based real-time Delphi [11].

### 1.4. Criticism

Early criticisms of the Delphi technique focused on weaknesses in the technique’s purported scientific rigor and questioned the ability of the questionnaires to provide reliable, representative results [6]. While researchers today may be more circumspect when it comes to making grand claims about the technique’s abilities, there remain serious limitations that must be considered when using the Delphi technique. As Bolger and Wright note, despite the developers’ initial intentions, social and psychological pressures remain a strong determinant of opinion change among participants, skewing poll results [5]. Furthermore, the use of the term “expert” has been questioned as imprecise, ineffective and exclusionary [12]. Often the term “expert” is used to designate those with professional qualifications, or who have researched the subject at hand, such as managers and academics, and not necessarily those with direct experience of the issue itself, such as service users at a hospital [12]. A final weakness of the Delphi model is the difficulty in defining and measuring consensus, the interpretation of which has changed repeatedly depending on the context and purpose of the study [1]. Nevertheless, the Delphi technique remains very popular as both a means of gathering information on a subject [9] and facilitating group discussion [3]. Grand claims of absolute objectivity and reliability notwithstanding, it remains an effective way to combine qualitative and quantitative assessments in a group setting.

### 1.5. Use in Development

Since its first increase in popularity in the late 1960s, the Delphi technique has seen widespread use in a number of different academic disciplines, as well as in commerce and public policy. In particular, the technique has seen widespread application in the fields of social forecasting, consensus interpretation of public health realities, and the formulation of public policy, especially in regards to health [9,3,12]. However, most of these studies have taken place among researchers in the Global North, with relatively few cases its adoption by researchers in the Global South [13]. Of the few studies that exist outside of Europe and North America, we identified two studies similar to our own that merit comparison: an economic evaluation of schistosomiasis treatment options in Kenya [13] and a study of occupational health research priorities in Malaysia [14]. The first, similar to our own study, adopts a highly modified Delphi approach in which individuals with experience with different schistosomiasis interventions were to make subjective judgments of the effectiveness of different treatments [13]. The study consisted of both local and foreign experts involved in public health initiatives in the country [13]. According to the researchers conducting the study, the reliability of the Delphi activity was limited by the number of experts with experience in each of the treatments as well as some difficulties in communication and ensuring anonymity [13]. Instead of representing a definitive overview of schistosomiasis treatment options, according to the researchers, it is better to see it as a first step in identifying priorities for future study.

The second study was conducted to identify the primary health risks affecting Malaysian workers, and featured respondents from government ministries, industries, professional organizations and universities [14]. The survey was conducted through two questionnaires in which priorities were ranked [14]. The researchers felt that the study was effective in identifying the top priorities for occupational health research, with a broad consensus across the sectors, though some sectoral biases were still detectable [14]. Importantly, the authors noted that the results of the study, both in the priorities outlined and the importance given to occupational health research itself, differed considerably from similar Delphi surveys conducted in countries in the Global North, in this case the United Kingdom, the Netherlands and Finland [14]. Thus, researchers should be mindful when using a technique primarily developed in the Global North in different social, political, and cultural contexts, and should be careful when making comparisons between the two regions.

**Figure 1:**
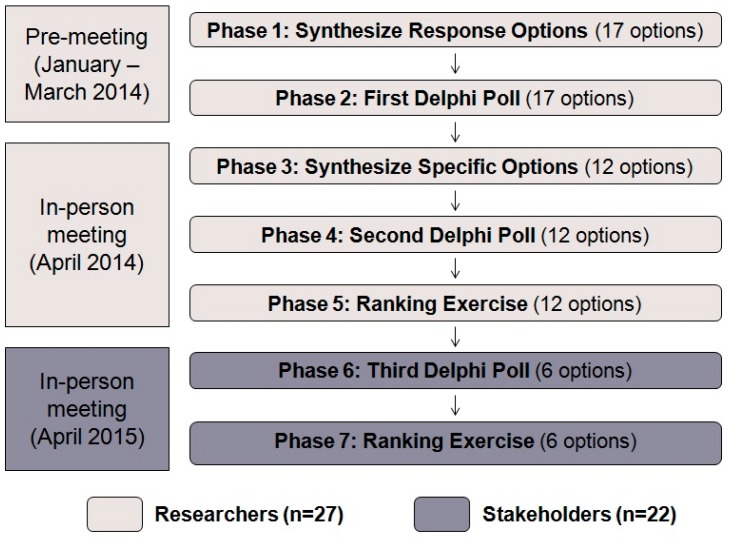
A flowchart of the Delphi exercise.

## 2. Delphi Methodolog*y*

We conducted a modified Delphi activity similar to the schistosomiasis study combining both anonymous polling and face-to-face group discussion (Figure 1). Similar to the article cited above, our study did not seek to make future predictions, but rather serve as a means of identifying and prioritizing future actions to address the social, economic, health and environmental effects of artisanal and small-scale gold mining (ASGM) in Ghana. Current policies regarding ASGM are insufficient, and there is urgent need in Ghana for determining national responses in terms of policy, legislation and interventions, which the Delphi poll aimed to clarify [15]. The goal was to select a handful of areas in which future study can be directed by assessing expert opinion on the subject, with the hope of shaping government policies on the issue.

As part of a three-year grant-funded project [15], participants involved with research or with experience on ASGM-related issues were selected from academia, government entities, and NGOs both from Ghana (n = 17) and North America (n = 10). Upon selection participants were organized into three working groups based on their area of expertise (Human Health, Natural Sciences and Social Sciences and Economics), with some individuals taking part in multiple working groups [16,17,18].

Unlike other studies we reviewed that used the Delphi method, the working group members were involved in the construction of the poll itself, democratizing control of the survey and increasing participant involvement. Using an Integrated Assessment approach [15], working group members had been collaborating for several months on discipline-specific reports summarizing extant and emerging research on ASGM-related issues [16,17,18] being published in this special issue of the International Journal of Environmental Research and Public Health (http://www.mdpi.com/journal/ijerph/special_issues/asgm). In the final section of these reports, they drafted possible “response options” or actions to address the social, economic, health and environmental issues associated with ASGM. For each of these response options, they included the cause or consequence of ASGM that the response option would address, the rationale for that response option, and potential actors and processes involved. At the end of the process, there were 14 potential survey response options from the Human Health working group, 13 from the Natural Sciences and 0 from the Social Sciences and Economics. 

In **Phase 1** (Figure 1) team leaders reviewed these response options and condensed them into 17 response options which were shared with participants via an online poll (SurveyMonkey.com). In **Phase 2**, participants were asked to rank the options in terms of benefit (broken down into the categories of economic benefit, environmental benefit, and benefit to people) and feasibility (broken down into the categories of economic feasibility, social/cultural feasibility, political feasibility, and implementation feasibility) on a four-point Likert-style rating scale (Figure 1). The values on the scale were *very low*, *low*, *high*, and *very high*. The scale purposely lacked a neutral point in order to elicit a firm position from the working group members. Descriptive statistics of the scores for each category of the poll were compiled and then shared with the participants on the first day (Day 1) of an in-person meeting in Accra in April 2014.

The level of specificity of the 17 response options in the first iteration of the poll was deemed insufficient for the purpose of establishing priorities. For **Phase 3,** each working group was asked to develop three to five *specific* response options (Figure 1). To do so, individuals first privately developed six to 10 specific response options and then shared them with their working group. Each working group then compiled individual members’ responses, generating a list of between 60 and 80 response options per group. From this list, they were asked to distill and prioritize three to five specific options to present to the entire team on Day 2 of the meeting. Working groups were asked to justify their specific response options based on the data in the working group reports, the benefit and feasibility scores from the poll, and their own expertise. 

**Table 1:** Response options for the first and second Delphi poll.

Response options for first Delphi poll
*It is recommended that...*
*A: ... leaders in Ghana engage in long-term planning and policymaking that addresses the unique challenges of ASGM.*
* B: ... social support for families in ASGM communities, particularly children and women, be increased.*
* C: ... employment opportunities available to persons in ASGM areas be diversified.*
* D: ... provision of basic services in ASGM communities (e.g., electricity, safe water, sanitation, health care) be increased.*
* E: ... Fair Trade Gold programs and initiatives be developed and implemented.*
* F: ... ASGM activities be physically separated from residential areas.*
* G: ... gold be obtained using mercury-free methods.*
* H: ... gold harvesting be conducted in a common, licensed facility with appropriate control technologies (e.g., mercury-capture, ventilation).*
* I: ... land reclamation be required after ASGM activities cease (e.g., returning lands to original contours or an improved state).*
* J: ... explorations for gold deposits be formalized and include assessment of heavy metals in surrounding ore of potential ASGM sites.*
* K: ... conditions to help ASGM miners register, regularize, and develop their mining activities be promoted.*
* L: ... legislation that governs ASGM in Ghana be revisited and revised if necessary, and effectively enforced.*
* M: ... a forestry initiative to reduce deforestation and land degradation associated with ASGM be implemented.*
* N: ... government entities provide educational campaigns and training centres for ASGM concession owners and miners, focused on health, safety, and environmental impacts.*
* O: ... ASGM miners be equipped with personal protective equipment (safety boots, hardhats, masks, hearing protection, etc.)*
* P: ... the government encourage research into the problems and benefits associated with ASGM, and that long-term implications for ASGM communities be prioritized*
* Q: ... the appropriate parties in Ghana sign the Minamata Convention and develop a National Action Plan (NAP) that includes mercury monitoring, and also helps link government agencies, policy makers with in-country scientists and the regional/international community.*
**Response options for second Delphi poll**
*It is recommended that...*
* A: ... a national framework for policy and planning implementation be established (i.e., taskforces, workgroups) that considers stakeholder input.*
* B: ... a national monitoring plan for mercury be established that includes inventories and abiotic media.*
* C: ... zoning requirements to separate mining from residences be enacted.*
* D: ... the Ghana Health Service increase health care access in ASGM Communities.*
* E: ... the government provide water, electricity, telecommunications, and sanitation in partnership with enterprises to ASGM communities and other affected communities.*
* F: ... registration of small-scale miners be increased by improving the process by, for example, reducing or eliminating fees and localizing registration.*
* G: ... local authorities in ASGM communities make special efforts to enforce regulations to prevent child labour and keep kids in school.*
* H: ... the Minerals Commission, EPA, miners, and universities embark on a study on pilot enforcement and implementation of current ASGM-related laws to assess their effectiveness.*
* I: ... ministries, local governments, and District Assemblies promote diversification of economic opportunities.*
* J: ... there be public and private support for education with ASG miners on ecological and human health risks, mercury and metals, mercury reduction strategies and business practices.*
* K: ... the royalties from the proceeds of mining be placed into a central account and directed towards improving health and environment of ASGM communities.*
* L: ... universities, the EPA, and the Minerals Commission explore and implement high-yield mercury free alternatives.*

**Figure 2:**
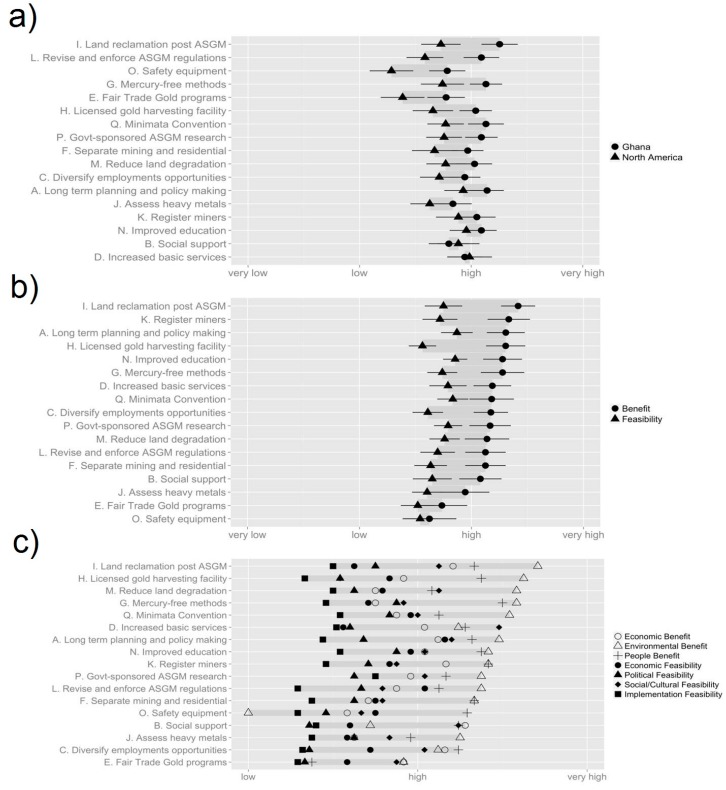
Delphi poll scores from the first Delphi poll, conducted among academics (n = 27) in March 2014. (**a**) Overall score for each option, grouped by region, sorted by difference; (**b**) Overall score for each option, grouped by benefit and feasibility, sorted by decreasing benefit; (**c**) Overall score for each option, grouped by topic, sorted by maximum benefit/feasibility.

**Figure 3:**
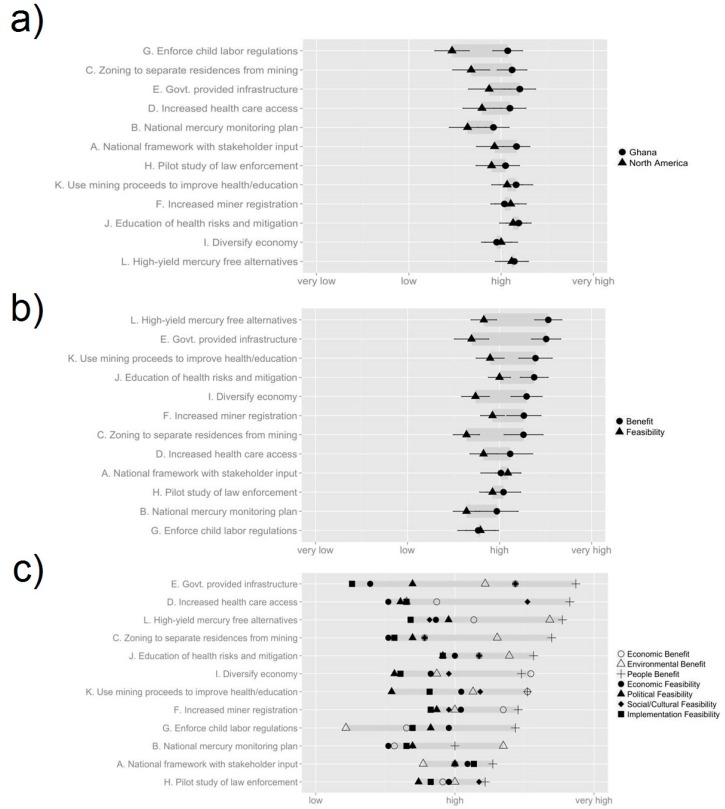
Delphi poll scores from the second Delphi poll, conducted among academics (n = 27) in April 2014. (**a**) Overall score for each option, grouped by region, sorted by difference; (**b**) Overall score for each option, grouped by benefit and feasibility, sorted by decreasing benefit; (**c**) Overall score for each option, grouped by topic, sorted by maximum benefit/feasibility.

**Figure 4:**
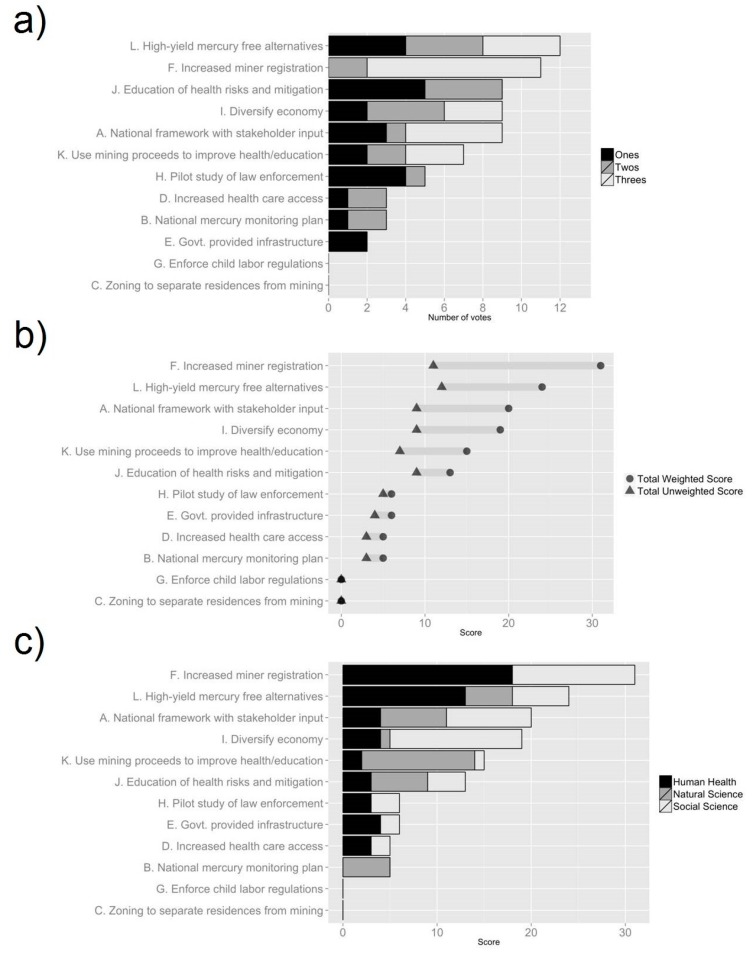
Results from sticky note exercise used to prioritize response options, conducted among academics (n = 27) in April 2014. (**a**) Number of votes for each option; (**b**) Weighted *versus* unweighted scores for each option; (**c**) Weighted scores for each option, grouped by workgroup.

**Figure 5:**
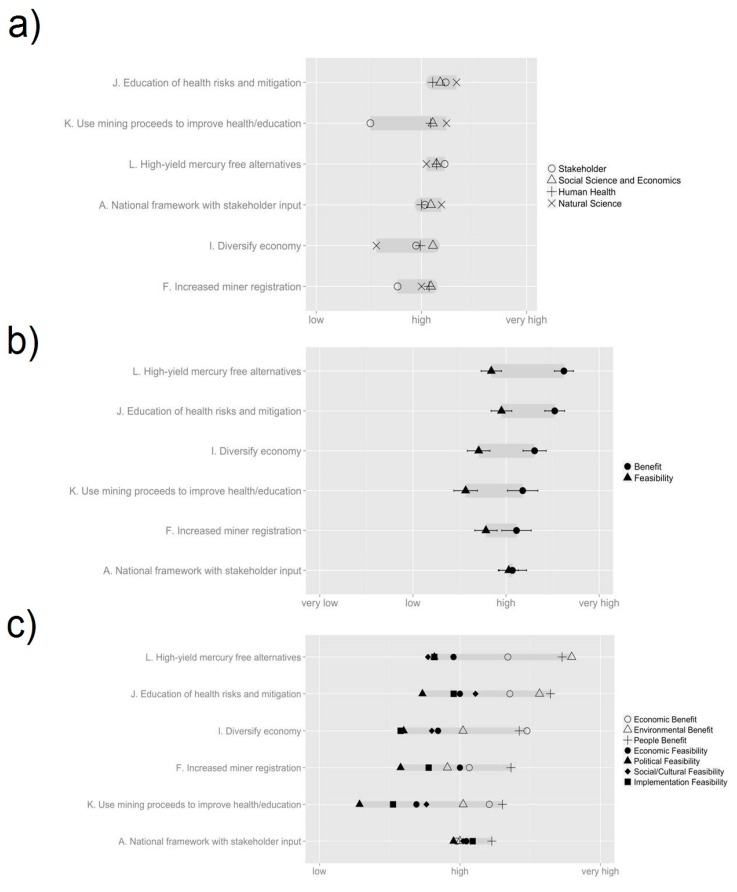
Delphi poll scores from the third Delphi poll, conducted among stakeholders (n = 22) in April 2015. (**a**) Overall score by workgroup; (**b**) Overall benefit and feasibility scores for all groups; (**c**) Benefit and feasibility score for each option grouped by topic.

**Figure 6:**
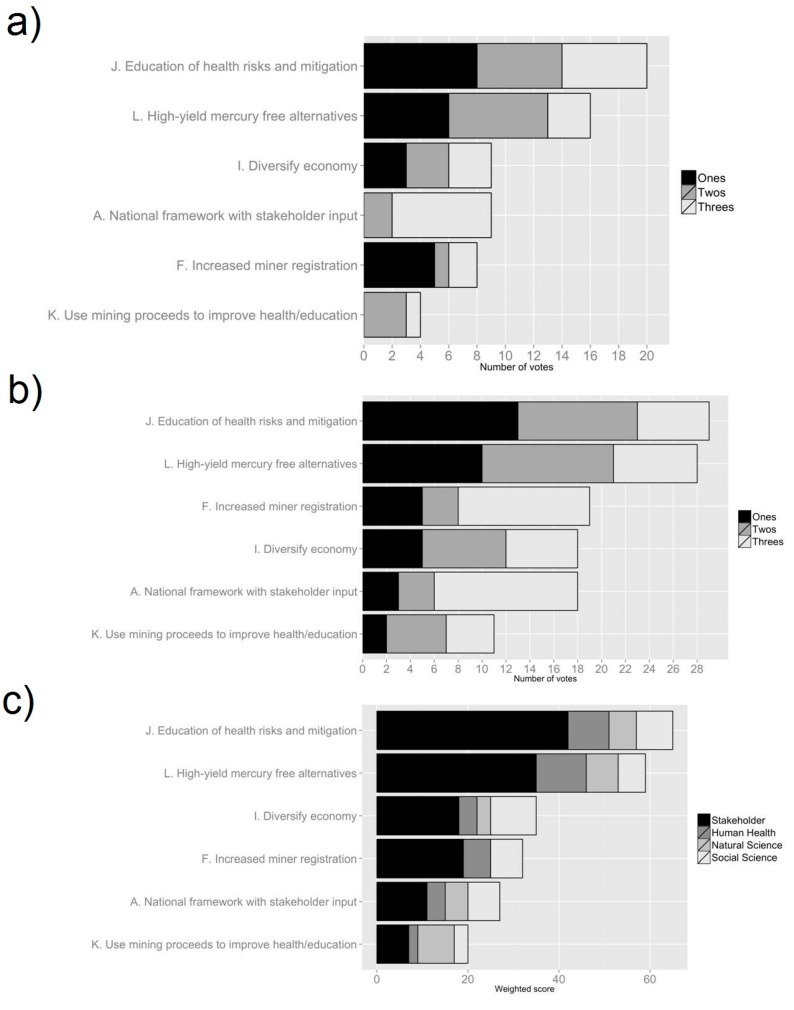
Results from sticky note exercise used to prioritize response options, conducted among academics (n = 27) in April 2014 and among stakeholders (n = 22) in April 2015. (**a**) Number of votes for each option; (**b**) Combined sticky note scores for all groups (academics and stakeholders); (**c**) Weighted scores for each option, grouped by workgroup.

Each working group developed six refined options and presented them to the team on the second day. The entire group discussed each response option after it was presented with the assistance of an experienced facilitator. The team leaders then condensed the 17 total specific response options into 12 specific response options by collapsing similar ideas among the groups. Once these 12 had been agreed upon, a second Delphi poll (**Phase 4**) was conducted with the same format as the first and with the new specific response options (Figure 1). The poll was conducted among the working group members present using a Microsoft Excel template that each individual submitted electronically to the project manager. Descriptive statistics of the scores for each category were shared among the entire group on the last day of the meeting (Day 3). 

To get a ranking of the 12 specific response options, on Day 3, each participant was given three different colored sticky notes and the options were written out on whiteboards (**Phase 5**, Figure 1). The team was asked to place their stickers under what they considered the first, second and third most important response options. These results were subsequently transcribed and represented graphically. 

A year later, a meeting was convened of relevant stakeholders representing local and national government, academia, NGOs, miners’ associations, and other organizations (Supplementary Tables). Study findings were presented to the stakeholders. Prior to the meeting, the six top response options (A, F, I, J, K, L) from the second round of the Delphi were determined via their consistently high scores on several metrics. These top six options were presented to the group, and in **Phase 6** (Figure 1), the stakeholders rated the options using a paper version of the same Delphi poll questions described for the first two Delphi polls. Participants also indicated a ranking of the top three most important options on this paper poll (**Phase 7**, Figure 1). These results were also transcribed and represented graphically. 

## 3. Results

In the first Delphi poll, respondents provided their initial perceptions of the 17 options previously identified and began to formulate policy priorities for ASGM communities. The response options for the first poll are shown in Table 1. The results of the first poll can be found in Table S2, Figure S1, and Figure 2. In the second Delphi poll respondents were to assess the benefits and feasibility of the 12 options that had been the developed from the original 17. The twelve options are listed in Table 1, and the poll results can be seen in Table S3, Figures S2 and S3, and Figure 3. In the first sticky note exercise, administered after the second Delphi poll, respondents were asked to rank their first, second and third preferred options, the results of which can be seen in Figure 4. The overall stakeholder poll and sticky note exercise scores can be seen in Figure 5 and Figure 6. Overall scores for the top six options across both the stakeholder and academic groups are shown in Figure S4. Individual comments on the options presented in all of the Delphi polls are presented in Tables S4–S6.

### 3.1. Results from the First Delphi Poll

Of the 18 options in Poll 1, the top two with the greatest benefit scores were **Option I** (that land reclamation be required after ASGM activities cease) and **Option K** (that conditions to help ASGM miners register, regularize, and develop their mining activities be promoted). The three with the greatest feasibility scores were **Option A** (that leaders in Ghana engage in long-term planning and policymaking that addresses the challenges of ASGM), **Option M** (that a forestry initiative to reduce deforestation and land degradation associated with ASGM be implemented), and **Option N** (that government entities provide educational campaigns and training centers for ASGM concession owners and miners, focused on health, safety, and environmental benefits) (Note, **Options M** and **N** were tied) (Figure 2b). The top three options overall were **Option A**, **Option N,** and **Option I** (Figure S1).

### 3.2. Results of the Second Delphi Poll

Of the 12 options in Poll 2, the three with the greatest benefit scores were **Option L** (Universities, EPA, the Minerals Commission explore and implement high yield mercury free alternatives), **Option K** (Royalty from proceeds of mining needs to be placed into a central account and directed towards improving health and environment of ASGM communities), and **Option E** (Government should provide utilities in partnership with enterprises to ASGM communities and other affected communities). The three options with the greatest feasibility were **Option A** (Establish a national framework for policy and planning implementation that considers stakeholder input), **Option H** (Minerals Commission, EPA, miners, universities. study on pilot enforcement and implementation to assess effectiveness of laws), and **Option J** (Public/private support education with ASGM on ecological and human health risks, Hg and metals, Hg reduction strategies and business practices) (Figure 3b). The three options with the greatest score overall were **Option J**, **Option L** and **Option K** (Figure S2).

### 3.3. Results of the Sticky Note Exercise

Unlike the Delphi polls, the results of the sticky note exercise showed a much greater degree of variability. First choices were highly concentrated with four options (**J, H, L, A**) accounting for 68% of respondents top choices (Figure 4a). The rest vary between 4 and 8%, and three options (**F, G** and **C**) receiving no first choices. When looking at the top scores, both weighted (1st choice = 3 points, 2nd choice = 2 points, 3rd choice = 1 point) and unweighted (each sticky = 1 point) we see a similar concentration (Figure 4b). In the case of the unweighted scores the top two choices (**L** and **F**) received 11 and 12 of the 72 sticky notes respectively (32% overall). For the weighted scores the disparity is more pronounced with the top two choices (**L** and **F**) receiving 31 and 24 of 144 points respectively (39% overall).

The option with the highest number of first choices was **Option J** (There should be public and private support for education of ASG miners on ecological and human health risks, Hg and metals, Hg reduction strategies and business practices). The option with the highest unweighted score was **Option L** (The universities, EPA and Minerals Commission should explore and implement high yield Hg-free alternatives). The option with the highest weighted score was **Option F** (Increase ASGM registration by improving the process) (Figure 4a,b).

The option with the highest weighted score for the *Human Health* workgroup was **Option F**. For the *Natural Science* workgroup it was **Option K** (Royalties from proceeds of ASGM need to be placed into a central account and directed towards improving health and environment in ASGM communities) and for the *Social Science/Economics* workgroup it was **Option I** (Ministries, local government, and District Assemblies should promote diversification of economic activities) (Figure 4c). 

### 3.4. Results of the Stakeholder Poll

At the meeting conducted one year after the initial Delphi meeting, stakeholders rated the six top response options (A, F, I, J, K, L). Figure S4 shows the overall score for each of the options across both the stakeholder and academic groups. **Options J** and **L** are rated the highest across all the groups. Figure 5a shows the preferences for the different groups, revealing that, of the six options, stakeholders rated **Option L** the highest and the **Option K** the lowest. Figure 5a,b show the relative benefit and feasibility of the six options. Across all the groups, **Option L** had the highest benefit while **Option A** had the highest feasibility. In general, the benefit of the options was perceived to outweigh their feasibility.

### 3.5. Results of the Stakeholder Sticky Note Exercise

The stakeholders also participated in a sticky note exercise of the six options provided. Figure 6a shows the voting patterns from the stakeholder group, revealing the most votes for **Options J** and **L**. They also had several number one votes for **Option F**. When the votes from natural, social, human health sciences are combined with the stakeholder votes, **Option J** and **L** maintain their top position, though stakeholders account for most of the voting (Figure 6b). Figure 6c shows the weighted scores for each of the groups. Once again **Options J** and **L** are the most desirable options.

## 4. Discussion

In both the first and second poll, there was little variation in the scores. Thus it is difficult to say that a consensus around which options to pursue was developed. However, analysis of these poll results do point to some broad trends which could be used to guide future action. 

The first of these trends is the remarkable uniformity for the different response options in both the first and second Delphi polls. In the first poll the standard deviation for benefit and feasibility scores are relatively small (σ = 0.207 and σ = 0.11 respectively), with benefit scores ranging between 2.63 and 3.42 and Feasibility scores ranging between 2.63 and 2.85. In terms of benefit respondents tended to score options higher in terms of Environmental Benefit (μ = 3.28) and lowest in terms of Economic Benefit (μ = 2.94). For Feasibility, scores were highest in terms of Social/Cultural Feasibility (μ = 2.98) and lowest for Implementation Feasibility (μ = 2.43) (Figure 2c).

For the second poll, both in terms of benefit and feasibility, there was little variation between the options (σ = 0.23 and σ = 0.14 respectively), with benefit scores ranging between 2.96 and 3.52 and Feasibility scores ranging between 2.52 and 3.1. In terms of benefit respondents tended to score options higher in terms of Benefit to People (μ = 3.5) and lowest in terms of Environmental Benefit (μ = 3.05). For Feasibility, scores were highest in terms of Social/Cultural Feasibility (μ = 3.04) and lowest for Implementation Feasibility (μ = 2.73) (Figure 3c). In all cases, respondents perceived the benefit of the options to be greater than their feasibility. In particular, options tended to score lower in political and implementation feasibility, suggesting the existence of institutional barriers preventing options from being realized (Table 1). In the respondents’ comments (Tables S4–S6) this was expressed, with respondents citing lack of trust between miners and the government and lack of political will to address the issues surrounding ASGM as key barriers to reform. Another important trend that emerged was the high level of similarity among the benefit scores (μ = 3.08, μ = 3.05, μ = 3.5). In some cases, a large discrepancy between the different benefits occurred. This suggests that in some cases respondents saw a trade-off between economic, environmental and social benefits.

While there tended to be high levels of agreement both between and within options, the same was not true of the Sticky Note Exercise. As discussed above, people's choices tended to be a lot more concentrated, with a few high scoring options emerging. Interestingly enough, despite the Sticky Note Exercise not being part of the standard Delphi technique, it was a lot more effective than the questionnaires in building a consensus, with responses clustering around two top options similar to the disaggregative policy Delphi described by Tapio [10]. Whereas in the questionnaires there was no limit on respondents ability to score options, allowing them to rank them more or less equally, in the Sticky Note Exercise they were more constrained, forcing them to prioritize. Thus, the Sticky Note Exercise proved more effective in highlighting future policy priorities. However, the multi-round Delphi survey gave respondents the ability to discuss and review all the options; hopefully providing greater depth and deliberation to respondents’ final decisions.

### 4.1. Benefits of the Exercise

The exercise proved a success in assessing the benefits of different options and developing policy priorities with expert input from diverse disciplines. In the second Delphi poll, three options were highlighted as having high feasibility and benefit; education programs about mercury health risks and reduction strategies (**Option J**), royalties from mining being directed towards health and education in mining communities (**Option K**) and exploring and implementing high yield Hg-free alternatives (**Option L**). All three options were seen as successful in combining environmental, economic and social benefits with high levels of feasibility (Figure S3). All three choices scored well in the Sticky Note Exercise, with **Option L** receiving the most first choices from *Natural Science*, **Option J** receiving the most first choices from *Human Health* and *Social Sciences/Economics* and **Option K** the top weighted score for *Natural Science*. However, the option that received the highest weighted score for all three workgroups was **Option F** (Improve registration for ASGM), an option which was seen during the second Delphi as having little environmental benefit and low implementation and political feasibility (Figure 3c). Thus, we should be careful in drawing too strong of a connection between participants’ responses in the Delphi and Sticky Note Exercise, with very different rationales and pressures possibly shaping people’s answers in the two exercises.

### 4.2. Criticisms 

Experience with the Delphi technique suggests it has a number of important benefits which serve to justify our use of it in this project. However, there are a number of important criticisms and limitations to consider both when analyzing our results and when considering future Delphi use. First and foremost of these is the use of the term “experts”. As discussed above, the use of the term can be exclusionary and privileges some forms of knowledge over others [12]. Our first panel was comprised of Ghanaian and North American academics, and our second panel included members of several stakeholder organizations. While respondents' decisions may be informed by conversations, they have had with small-scale miners in the field, in the future more effort should be made to incorporate their direct involvement in the Delphi process. 

Another important limitation to consider is the overly quantitative nature of Delphi. While the point system used is effective in surveying a large group of individuals, organizing information and observing statistical trends, it does have a tendency to simplify responses and ignore nuance. While respondents were asked to rate options based on different feasibility and benefit criteria, little information is available to understand the respondents' rationales. Though a comments section was provided, only some respondents choose to elaborate on their decisions. Much discussion did occur informally between polls in the workgroup and team sessions; however, these sessions were not recorded, leaving little than can be incorporated into the final report. Past Delphi polls have been conducted in which respondents are asked to include qualitative rationales to defend their quantitative responses, which are then shared with the rest of the group [5], an option which it might be advisable to pursue in the future. In addition, by formulating priorities by looking at those options with the highest mean scores and lowest standard deviations, outliers are often missed and differences between respondents are erased. As Tapio writes, often cases of discord rather than consensus prove the most enlightening [10], an idea not emphasized in this study.

A final criticism to consider is the role social pressures may have played in shaping respondents’ decisions. In theory the anonymous polling of the Delphi technique is supposed to limit these pressures [2]; however, in many cases they continue with participants over-valuing their own opinion, undervaluing those of others, and conforming to those views perceived as dominant [5]. Thus it is important to consider what role these pressures played in shaping group opinion, especially during face-to-face interactions such as the between poll sessions or the Sticky Note Exercise.

## 5. Conclusions

Assessing the benefits of different options and identifying priorities is important when developing future policy. The latter is especially important in situations where resources are limited and a number of options must be looked at to determine which will be the most effective. The timing is ideal for this exercise for several reasons. First, in 2013, the member nations of the United Nations Environment Program (UNEP) agreed on the Minamata Convention on Mercury. The Convention has a stand-alone article (Article 7) and a dedicated Annex (Annex C) concerning the ASGM sector. Countries with significant ASGM activities, including Ghana, are required to create a public health strategy for ASGM communities, and take specific measures to protect vulnerable populations such as children and women of childbearing age. In addition, the Convention encourages Parties to cooperate in education, outreach and capacity-building initiatives specific to ASGM (7.4B), as well as enhance public health measures to address mercury pollution (Article 16), especially for vulnerable groups (16.1A). Strengthening of institutional and health professional capacities is also specifically mentioned (16.1D). Many of these focal areas emerged in our Delphi polling exercise. At the country level, ASGM is addressed in Ghana’s Minerals and Mining Act, 2006 (Act 703). While many miners are formally registered, approximately 80% of ASGM miners are unregistered and thus mining illegally (personal communication with Ghana Minerals Commission, 6 August 2014). In 2013, in an effort to legitimize the sector, the President of Ghana initiated an Inter-Ministerial Task Force on Small-Scale Mining. Also, the National Development Planning Commission (which reports to President of Ghana) has recently identified ASGM as a sector of major economic, social and national security concern that thus demands rapid policy action. The results of this Integrated Assessment [15] and identification of response/policy options via Delphi polling (current paper) were shared with representatives of the aforementioned programs, and other stakeholders.

With respect to the aforementioned international and national endeavors, and given the situational context of the work, Delphi style polling can be used as a quick way to gauge the opinions of a select group with a high degree of expertise. In the case described here, multi-round Delphi polling was effective in exploring a number of options surrounding small-scale gold mining in Ghana, and was able to highlight which options to pursue further with results being shared with relevant parties. That being said, highlighting priorities in of itself is not useful. More research must be done surrounding these options, to further explore the benefits and challenges associated with each. Future researchers and policy makers must be aware of the limitations of Delphi type surveys if they are not to recreate them. In particular, greater efforts must be made to include the views of miners in assessing options and formulating and implementing policy to address the exclusionary effects of the Delphi technique. In the same vein, research must move beyond the simplistic quantification systems of the polling process but rather look at the options described in all their complexity. The Delphi process is a very useful starting point, to what is and what should be, a much longer and more in-depth discussion.

## Supplementary Files

Supplementary File 1
