# Supplementary material for: Identification of Response Options to Artisanal and Small-Scale Gold Mining (ASGM) in Ghana via the Delphi Process
Source: Int J Environ Res Public Health. 2015 Sep 10;12(9):11345–63. doi: 10.3390/ijerph120911345 (PMC4586679; doi:10.3390/ijerph120911345)
Supplement: Supplementary File 1 [file ijerph-12-11345-s001.pdf]

Supplementary

Identification of Response Options to Artisanal and Small-Scale Gold Mining (ASGM) in Ghana via the Delphi Process

---

Table S1. Institutions represented at stakeholder meeting.

**INSTITUTION/ORGANIZATION**
Council for Scientific and Industrial Research-Science and Technology Policy Research Institute (CSIR-STEPRI)
Natural Resources Defense Council (NRDC)
University of Mines and Technology (UMAT)
Council for Scientific and Industrial Research-Water Research Institute (CSIR-WRI)
National Development Planning Commission (NDPC)
Ghana National Association of Small Scale Businesses
Tarkwa Chamber of Commerce and Industry
Accra Mining Network
Friends of the Nation
Institute for Development Studies, University of Cape Coast (IDS,UCC)
Ecological Restoration
Chamber of Mines
Ministry of Health Korle Bu Teaching Hospital (KBTH)
Wassa Fiase Traditional Council
Artisanal and Small-Scale Miners (ASM) Africa Network
McGill University
University of Michigan
McGill University
Solidaridad West Africa
Ghana Environmental Protection Agency (EPA)
Ghana Health Service
Wassa Association of Communities Affected by Mining (WACAM)
University of Ghana
Ministry of Land and Natural Resources
Ministry of Environment, Science, Technology, and Innovation
Tarkwa Municipal Assembly

Table S2. Results from the first Delphi poll.

Options	Economic Benefit	Environmental Benefit	Benefit to People	Mean Impact Score	Economic Feasibility	Social/Cultural Feasibility	Political Feasibility	Implementation Feasibility	Mean Feasibility Score	Overall Mean Score
**A**	3.12	3.48	3.32	3.31	3.16	3.20	2.68	2.44	2.87	3.06
**B**	3.28	2.72	3.24	3.08	2.60	3.24	2.36	2.4	2.65	2.83
**C**	3.16	3.12	3.24	3.17	2.72	3.04	2.36	2.32	2.61	2.85
**D**	3.04	3.24	3.28	3.19	2.56	3.48	2.6	2.52	2.79	2.96
**E**	2.93	2.92	2.38	2.74	2.58	2.86	2.33	2.29	2.52	2.61
**F**	2.70	3.33	3.33	3.12	2.75	2.79	2.63	2.38	2.64	2.85
**G**	2.75	3.58	3.50	3.28	2.71	2.92	2.88	2.46	2.74	2.97
**H**	2.92	3.63	3.38	3.31	2.83	2.54	2.54	2.33	2.56	2.88
**I**	3.21	3.71	3.33	3.42	2.63	3.13	2.75	2.50	2.75	3.04
**J**	2.63	3.25	2.96	2.94	2.58	2.83	2.63	2.38	2.60	2.75
**K**	3.17	3.42	3.42	3.33	2.83	2.88	2.71	2.46	2.72	2.98
**L**	2.88	3.38	3.13	3.13	3.04	2.79	2.67	2.29	2.70	2.88
**M**	2.75	3.58	3.08	3.14	2.79	3.13	2.63	2.5	2.76	2.92
**N**	3.04	3.42	3.38	3.28	2.96	3.04	2.88	2.54	2.85	3.04
**O**	2.58	2.00	3.29	2.63	2.75	2.67	2.46	2.29	2.54	2.58
**P**	2.96	3.38	3.17	3.17	2.75	3.04	2.63	2.75	2.79	2.95
**Q**	2.88	3.54	3.13	3.18	2.96	3.00	2.83	2.54	2.83	2.98

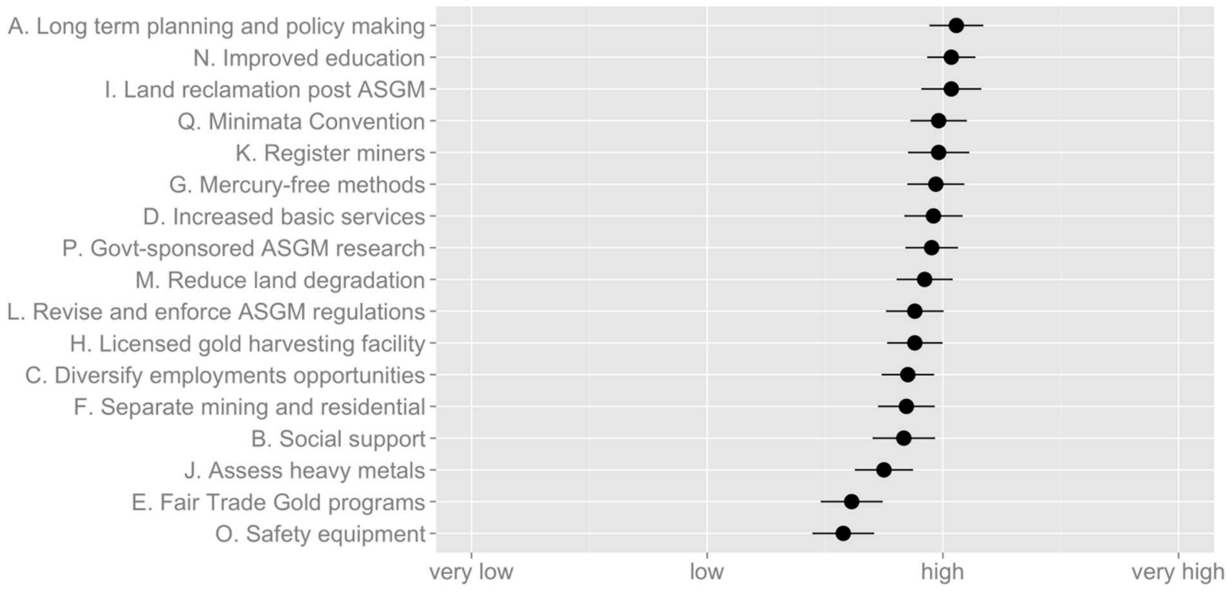

Figure S1. Overall score for each option, sorted by score, from the first Delphi poll.

Table S3. Results of the second Delphi poll.

Options	Economic Benefit	Environmental Benefit	Benefit to People	Mean Impact Score	Economic Feasibility	Social/Cultural Feasibility	Political Feasibility	Implementation Feasibility	Mean Feasibility Score	Overall Mean Score
**A**	3	2.81	3.24	3.02	3.1	3.14	3	3.14	3.1	3.06
**B**	2.59	3.32	2.96	2.96	2.5	2.68	2.68	2.64	2.63	2.76
**C**	2.77	3.32	3.68	3.26	2.5	2.77	2.68	2.59	2.634	2.9
**D**	2.91	2.73	3.82	3.15	2.55	3.5	2.59	2.64	2.82	2.96
**E**	3.46	3.18	3.86	3.5	2.46	3.41	2.73	2.32	2.73	3.06
**F**	3.32	2.96	3.43	3.23	3.05	2.96	2.86	2.86	2.93	3.06
**G**	2.64	2.27	3.41	2.77	3	2.68	2.82	2.68	2.8	2.79
**H**	2.86	3	3.19	3.02	3	3.14	2.76	2.86	2.94	2.97
**I**	3.62	2.91	3.55	3.35	2.82	3	2.55	2.64	2.75	3.01
**J**	3.14	3.36	3.55	3.35	3	3.18	2.91	2.91	3	3.15
**K**	3.55	3.09	3.5	3.38	3.05	3.19	2.57	2.81	2.91	3.11
**L**	3.14	3.67	3.76	3.52	2.86	2.81	2.95	2.67	2.82	3.12

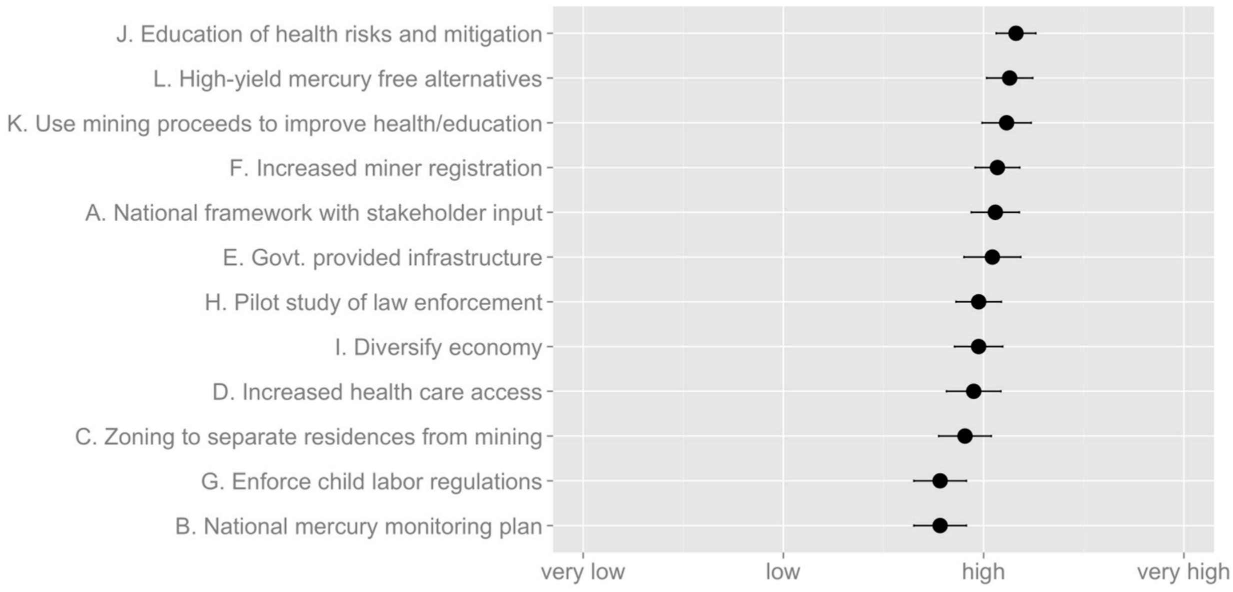

Figure S2. Overall score for each option, sorted by score, from the second Delphi poll.

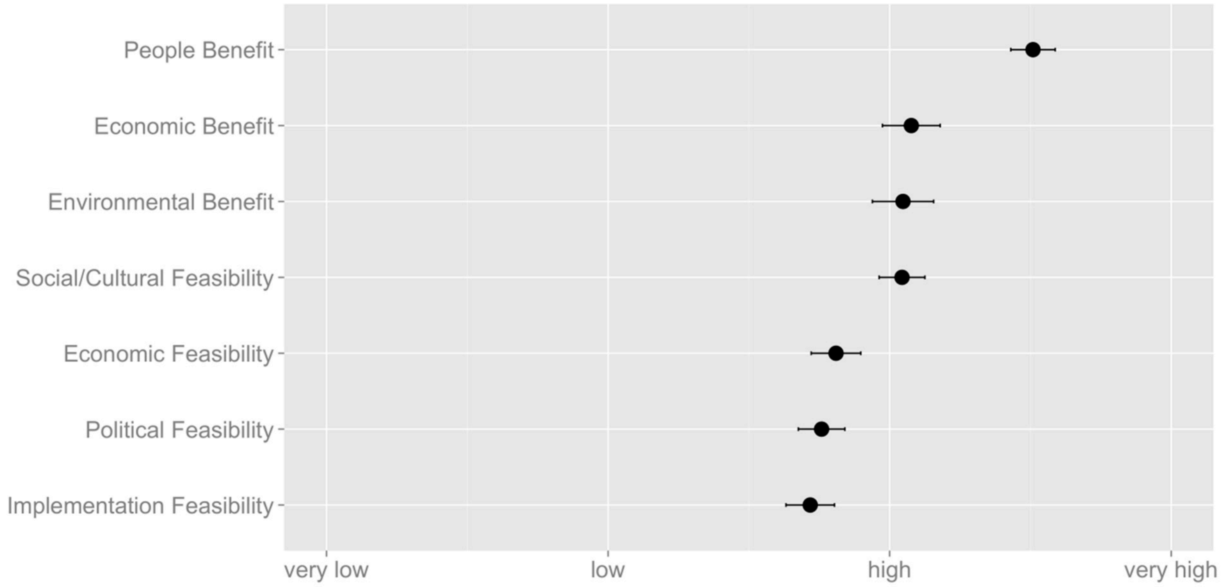

Figure S3. Score for each benefit/feasibility topic, from the second Delphi poll.

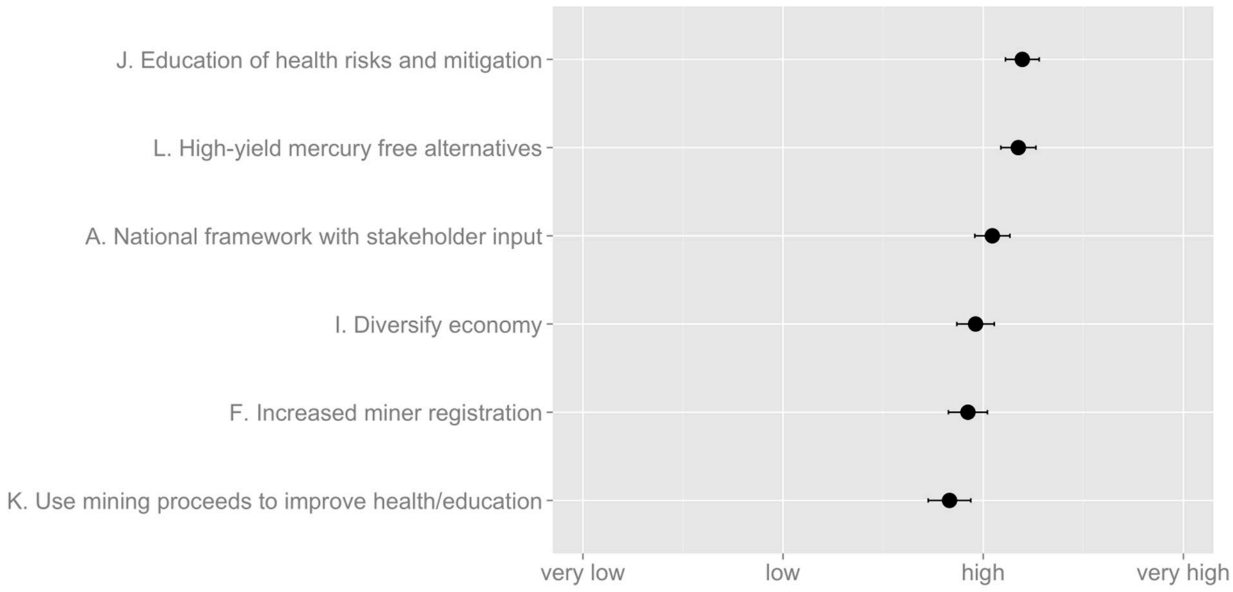

Figure S4. Overall score across the three Delphi polls for six options across both the stakeholder and academic groups.

Table S4. Respondent comments from the First Delphi Poll.

**Option A.**
I am not sure how this option differs in substance from some of the other more specific options that follow, regarding providing more assistance to miners with formalization and mineral rights, sorting out the mining code, providing more social services, etc. I am not a planning expert, so forgive me if this is just ignorance on my part; I don't understand the actual substance of that option. However, generally speaking, long-term planning and policy that takes this sector seriously will be a huge benefit.<br>It is important for define what we mean by engagement or better still the meaning standard of engagement so as to satisfy both parties.<br>The timescale is important. If done properly, the benefits will be realized but may be realized on the order of decades. My response to this question focused on near/medium terms (*i.e.*, 5–10 years).<br>I find this question difficult to answer in this format. Impact levels may depend on the kinds of policies in question and whether and how they are enforced. Some policies may have economic impacts but null or negative environmental and viceversa. There is also some overlap among the variables in questions, e.g., “Benefit to the people” may include benefit from economic growth or environmental regulation (e.g., water quality).<br>My perception on the feasibility is not based on specific knowledge of the situation in Ghana but more based on a perspective from other countries that are dealing with ASGM issues and the social and political challenges that need to be met.<br>The challenge that may arise is the issue of trust between Government (including its agencies), Mining companies and ASGM.<br>Need better understanding of current political situation and will to best answer the feasibility.<br>Obviously if such policies haven't been adopted and implemented yet, it is because of economic, social, political, and logistic constraints. So unless the latter are well understood and realistic strategies are devised to remove them, feasibility will remain low. But it will be necessary to identify the individuals and institutions who have a mandate and leverage to address such challenges.

Table S4. *Cont.*

**Option B.**
I am not entirely clear what social support is envisioned (healthcare, education, regulations against child labor?), and depending on the kind and quality of support, impact on each of those dimensions will be different.<br>What does social support for families in ASGM communities mean?<br>again, feasibility relative to those various domains depends on the type of "support".
**Option C.**
May have some impact but gold mining is a very attractive and profitable option compared to almost any other rural economic activity.<br>Alternative sources of employment in mining areas are probably very few. This will be a big challenge.<br>Even if there is diversification, it does not mean that there will not be ASGM if present prices prevail<br>Research shows that some efforts to diversify income in ASGM areas have not been very effective or acceptable. Once again, benefits to people may vary according to the relative profitability of different activities and impacts may vary according to the costs (in cash, labor, *etc.*) and resources needed (e.g., wood, water, *etc.*).<br>Environmental benefit depends on the types of employment diversification options.<br>These could be feasible—but wouldn't necessarily be feasible—depending on the local employment options<br>As above, feasibility will vary depending on what is being proposed as alternative employment or livelihood source.<br>Though preferable, it's my conviction that it doesn't seem to be a priority to the Government. Government focus over the years has been to stop the activity of the persons involved but not to provide alternative economic activities. There is actually no political will.
**Option D.**
There is always a huge econ and env benefit from improved water supply and sanitation!<br>These would be very beneficial to ASGM communities but it is very unclear whether they would be feasible in terms of costs, political interest, or implementation.
**Option E.**
I note that the group Solidaridad has already tested the idea of fair trade gold in Ghana. Maybe this option should be described as evaluate FT initiatives in Ghana and determine if they hold promise for expansion.<br>I do not understand the fair trade gold programmes but since the system requires me to answer before I can progress and I do not have an option of Not Applicable, I have selected these options just to allow me progress.
**Option F.**
Economic impact may be negative if people need to pay for daily transportation to separate mining areas. Or they may need to walk long distances, adding to the physical labor burden. In the latter case the separation will have negative benefit to the people.<br>Could separation of ASGM activities and residential areas lead to a larger are of land being degraded or altered from human activity/disturbances? (But human health benefits are valuable.)

Table S4. *Cont.*

**Option G.**
This will have a high economic impact because other recovery methods can be much better than mercury in terms of gold yield.<br>The economic impact may be negative if the alternative method is more costly, in which case it would not be a "benefit". It I am not sure how to answer this question, because it may be a significant impact but not a "benefit".<br>This needs serious commitment to help miners transition. I am not sure where the government stands right now in terms of political will.<br>If there are practical mercury-free methods that could be used by ASGM, this is a good option. I'm not aware that such methods are available in Ghana.<br>New methods currently exist such as the use of borax.
**Option H.**
Environmentally, great idea—but in practice, not feasible.<br>it all depends on how the facility is managed and how and by whom controls are exerted.<br>The idea of a common processing facility is one that has been tried many times in various locations around the world with wildly varying outcomes. The success very much hinges on the trust the miners have in the processors (often not much, with good reason), and on the system devised for assessing and sharing profits from the material produced. I am not dismissing this option, but perhaps a better way to describe it is to explore the option or pilot the approach, in order to figure out will work in the local existing economic system and culture. By the way, under no circumstance should such a facility use mercury.
**Option I.**
Economic and human benefits may be long-term and/or indirect.<br>Returning land to an improved state has been done already in many ASGM sites (not necessarily original contours but improved state). So this is quite feasible if there is political will to require it.<br>Who would monitor this reclamation?
**Option J.**
This will have a significant environmental impact because with better exploration, ASGM miners can have more targeted operations, with less land disturbance (theoretically anyway).<br>It will have a negative impact on ASGM unless there is some fair way of distributing concessions.
**Option K.**
This option presupposes that the laws/regulations are already adequate to address the needs of small scale miners, and that what is needed is help with implementation and enforcement. During the discussions in Accra, I would want to confirm with your team why they believe this is true. If not, perhaps the options should be expanded to include the idea of revising existing law to better suit ASGM conditions. Also, regarding the issue of mineral rights, which is a concept central to implementing all of these options, I would also want to confirm that there are areas available in Ghana for ASG miners to obtain mineral rights. I have heard anecdotally from people working in other countries that most workable areas in the entire country already have had the mineral rights assigned, and there is no "free land" for assignment to ASG miners. Since Ghana has such a long history of mining, I wonder if this is the case in Ghana as well. And if so, what is the relationship between miners and these mineral rights holders.<br>Depending on the circumstances, the area of study of determining and (re-)assigning mineral rights in a way that is beneficial to ASG miners could itself be a broad response option.<br>Currently I believe one of the constraints is that prices offered outside the formal system are higher, which means that economic benefits of formalization would be negative.<br>Environment impacts will be long term.

Table S4. *Cont.*

**Option L.**
See comment on Option K.<br>As in the case of policies, the impact of legislation depends on whether and how it is translated into enforceable regulations.
**Option M.**
Impact will depend on how the initiative is implemented.<br>How would the necessary oversight be funded?
**Option N.**
This broad option seems to assume a government run, top down approach to education. Often miner to miner exchange is more effective than top down education by the government. Also, education centers do not have to be government run per se, in Kevin Telmer's project in Burkina Faso right now, they have built a functioning processing center in a community that serves the processing needs of the community but also serves as a training location where miners from other communities come to watch, and get information. So, I would recommend making the broad option more broad to include all kinds of formulations for education and info exchange.
**Option O.**
Could be broadened to cover occupational safety generally (including accident prevention, which I know is a focus of some folks on your research team).<br>Feasibility is tied to how this would be financed, implemented, and enforced.<br>PPE use even in formal sector is low. ASGM sites implementation will be a very big challenge.
**Option P.**
Miner organizations and local communities should be active partners in the research rather than targets.<br>Outside funding from donor countries would help.<br>I supposed it would be feasible if funding is available, not sure whether and where from.
**Option Q.**
The issue of "who is in charge" has been a big barrier to creation of the NAP in other countries. Perhaps your multidisciplinary team would have some insights for the government about what a broad-based multi-stakeholder consultation should look like for the creation of the NAP. One response option that seems to be missing from the collection: the option to support and empower miners more generally through the small scale mining associations. I know some such associations already exist in Ghana, but how can they be strengthened to (1) provide miners with a strong collective voice in dealing with government on ASGM rights, the NAP formulation, *etc.*, and (2) provide a conduit for information and technology sharing? Miners need to be central partners in all of the activities you have described, so empowering SSMAs is one way to facilitate the participation of these critical stakeholders.<br>It's not clear to me why it hasn't been signed already.<br>This is not the first time the use of mercury in artisanal mining has been banned in Ghana. The overall cumulative effect is the upsurge of black market trade in mercury even under the current dispensation where the ban has been lifted so that ASGM operators can legally acquire mercury for their business.<br>there might be reasons why that hasn't happened yet, so those factors (and potential constraints) should be examined and addressed, so feasibility hinges on whether and how that can be done.

Table S5. Respondent comments from the second Delphi Poll.

**Option A.**
Interministerial.<br>Such programs already exist, and thus need to be strengthened and promoted.<br>The policies or legislation should take into account some of the regional treaties like the ECOWAS mining Directive.<br>“Easy” to implement, but results may not come quickly.<br>Necessary and useful, but prone to political infighting.<br>Some of the specific menu options will be easier to implement/more feasible than others.
**Option B.**
My scores seem low but this activity is called for in the Minamata Convention and will be required to assess Treaty efficacy.<br>May be a low priority for government.<br>Documenting Hg use/imports will be quite difficult since much of it is within the black market and/or not licensed.
**Option C.**
Hard to imagine that people will move their homes to achieve this; will need great motivation to do so.<br>Easy for new mining communities—but more difficult for established communities.<br>Does not seem very practical based on discussion.<br>There should be separete zoning requirements or processes for existing *vs.* new mining sites.<br>Social/cultural feasibility depends on existing *vs.* new mining sites.
**Option D.**
Lack of healthcare personnel to render this service.<br>Will require substantial funding.<br>Beneficial to ASGM communities but GHS may not prioritize these communities.
**Option E.**
Which enterprises?<br>How will this be funded and what incentives or drivers exist for this.<br>Very difficult but will have one of the greatest impacts on people and environment.
**Option F.**
Using legitimate organizations and trusted leaders within communities.<br>The fees currently being charged for registration is very low, however people are not registering. What needs to be done is provide some form of incentives such as Government guarantee loans for them or waiver on some of taxes.<br>High benefit to miners but low political will to do this, in a serious way (my suspicion).
**Option G.**
What is a special effort? Effort isn’t equivalent to outcomes.<br>Not very feasible to enforce; likely to meet community resistance.
**Option H.**
Funding available thru Min Convention.<br>With outside funding and core research team, this will likely be feasible; since the work is a pilot the benefits will (at first) be moderate and localized though potential for great benefit exists.

Table S5. *Cont.*

**Option I.**
In ways that address the diverse needs of different types of miners, with input by miners themselves.<br>Economic diversification is probably best initiated from the village-level. Perhaps an option like micro-financing would be something the state could promote.<br>This has been noticed to be the effective way of solving the current menace. Though high benefits, no effort on implementation.<br>Such a program will require investments, and not clear how such funds will be raised.<br>This hinges on development and passage of local content policy on mining.<br>Concentration should be on natives.<br>Promotion activities still unclear.<br>I suspect low political will for this option.
**Option J.**
Implement Free Compulsory Universal Basic Education as per constitution.<br>Parts of this are higher priority (e.g., reduction strategies) than other parts.
**Option K.**
Should not be a centralized account.<br>It should be implemented like the GETFund and RoadFund.<br>May be difficult to redirect funding if expectations are that funds will be spent elsewhere.<br>Likely to be objections from the central govt for local collection of money.
**Option L.**
Wording should include reduced Hg techniques as well. Transitioning from the current situation to zero-Hg is a big leap and one that is likely less feasible.<br>May yield great benefit to the miner if gold yields are increased, and also result in reduced Hg pollution.<br>Assuming such technologies exist and are available.<br>Very feasible! A fundable project!<br>Exploring for high-yield Hg-free alternatives would have a higher feasibility, but implementation may have a lower feasibility.

Table S6. Respondent comments from the stakeholder Delphi Poll.

**Option A.**
Benefits are low initial but would increase as their impact or work kicks in. Capital for implementation, political will may stand in the way.
**Option F.**
Any process without an online process would not yield any benefits in today's day and age.
**Option I.**
Political will would be needed for this to be successful. But this is non-existent
**Option J.**
Viable but the political will may not be available.
**Option L.**
Add civil society and miners to option L. Look at what else has already been done in Ghana and elsewhere and focus instead on implementation. Look at Artisinal Gold Council's work. For example: Processing facility in Damo, Burkina Faso. Look at miners in Ghana already processing without mercury ex. Using shaker table. Don't focus only on thermol retort (which can be complicated to use—see AGC guide) and Sikobokyia (direct smelting). See all options and focus on implementation.
**General**
Questions are not very concise. They leave a lot of room for subjective interpretation.<br>Education and awareness for the public and private stakeholders are essential for the ASGM action plan development and implementation. Humans and the environments are winners.<br>Mercury-free alternatives / best available techniques (BAT) are welcome to rid the environment and protect the human health from mercury emissions.<br>There should also be efforts to involve the private sector regarding the diversification of the economy. The focus should not only be in the government.<br>Promotion of voluntary ASM standards like Fair mined(?) Gold Standard (ARM) which presents direct economic benefits through premiums to miners who adhere to these criteria. These standards include Labor/Environmental and social criteria.

© 2015 by the authors; licensee MDPI, Basel, Switzerland. This article is an open access article distributed under the terms and conditions of the Creative Commons Attribution license (http://creativecommons.org/licenses/by/4.0/).
